# Supplementary material for: Growth phase influences virulence in Candidozyma auris systemic infection models
Source: mSphere. 2025 Dec 5;10(12):e00767-25. doi: 10.1128/msphere.00767-25 (PMC12724193; doi:10.1128/msphere.00767-25)
Supplement: Supplemental figures — Figures S1 and S2. [file msphere.00767-25-s0001.pdf]

Supplemental Material: Growth phase influences virulence in *Candidozyma auris* systemic infection models

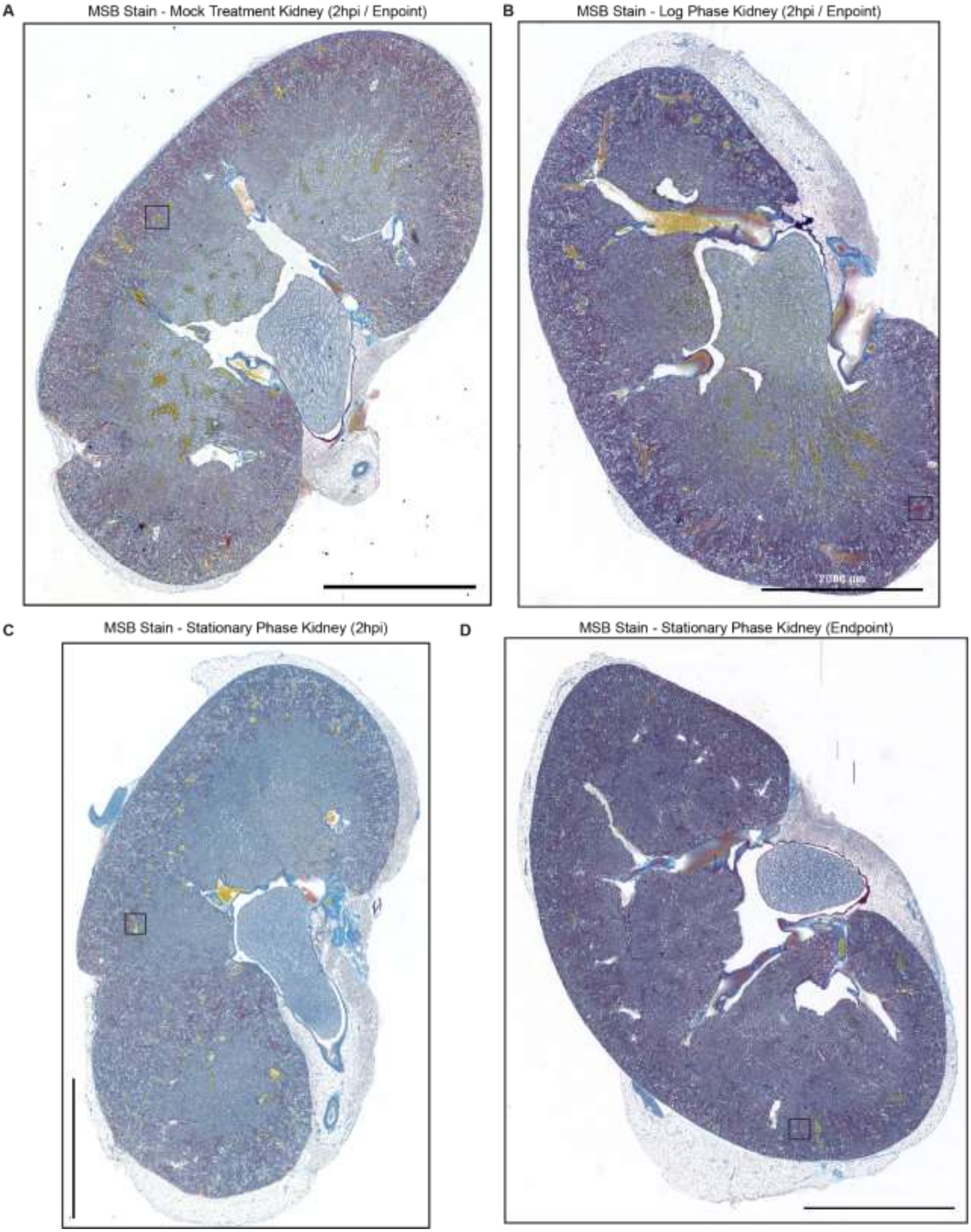
A
MSB Stain - Mock Treatment Kidney (2hpi / Enpoint)
B
MSB Stain - Log Phase Kidney (2hpi / Enpoint)
C
MSB Stain - Stationary Phase Kidney (2hpi)
D
MSB Stain - Stationary Phase Kidney (Endpoint)

Supplemental Figure 1: Related to Figure 3. (A-D) Full-size images of Martius Scarlet Blue staining of kidney sections from mice after mock treatment (A), or high-dose infection with *C. auris* in log phase growth (B), or stationary phase growth at a matched timepoint of 2 hpi (C), or post-mortality (D) (scale bars 2000 μm). Images are representative of 2 animals per cohort. Box indicates region expanded in Fig 3.

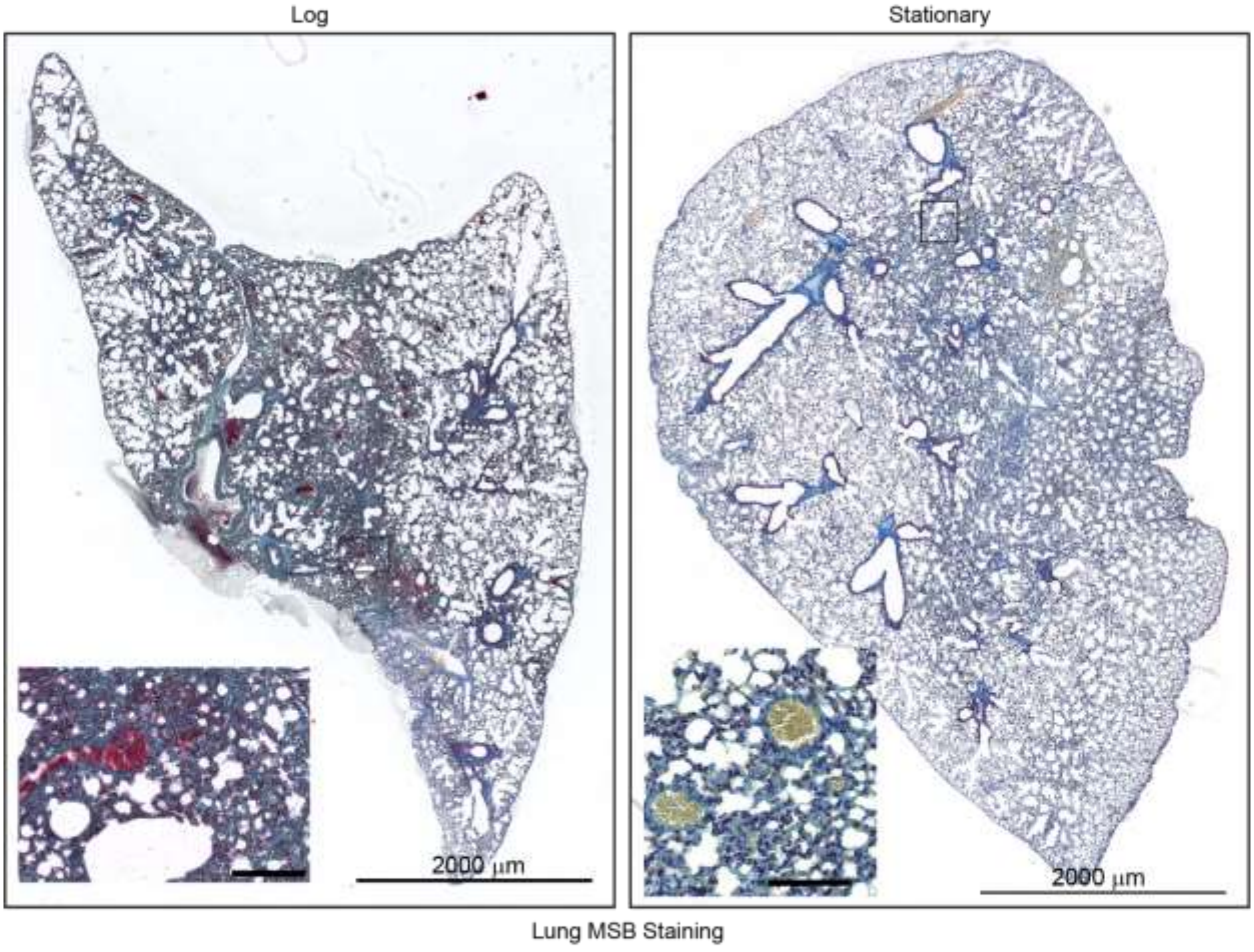

Supplemental Figure 2: Related to Figure 3. Representative MSB staining of lung sections from mice in high dose cohorts infected with log phase (left) or stationary phase (right) *C. auris* at time of mortality. Boxes show a magnified inset, showing Fibrin-positive erythrocyte-rich regions in log

phase *C. auris*-infected lungs or Fibrin-negative erythrocyte-rich regions in stationary phase *C. auris*-infected lungs (scale bar 100 μm). Images are representative of 2 animals per cohort.
